# Comment on “Renal Protective Effect of Sirtuin 1”

**DOI:** 10.1155/2015/780903

**Published:** 2015-09-20

**Authors:** Kultigin Turkmen

**Affiliations:** Division of Nephrology, Department of Internal Medicine, Meram School of Medicine, Konya Necmettin Erbakan University, 42090 Konya, Turkey

I read the review article written by Dong et al. [1] regarding the renal effects of sirtuin 1 with great interest. The authors described the harmful effects of advanced glycation end products on podocyte apoptosis via increasing Bcl-2 protein secondary to forkhead box O4 (FOXO4) acetylation in diabetes mellitus according to Chung et al.' findings. However, Chuang et al. [2] demonstrated that the apoptosis of podocyte was associated with the expression of a proapoptosis gene, Bcl-2-like 11 (Bcl2l11), not antiapoptotic Bcl-2. To date, a total of 25 Bcl-2 related genes have been identified. Among them, Bcl-2, Bcl-xL, and Bcl-w are antiapoptotic; however, BAD, Bax, Bak, and Bcl2l11 are proapoptotic [3]. Hence, researchers should consider this important knowledge to avoid misunderstanding.

## References

[1] Dong Y. J., Liu N., Xiao Z. (2014). Renal protective effect of sirtuin 1. *Journal of Diabetes Research*.

[2] Chuang P. Y., Dai Y., Liu R. (2011). Alteration of forkhead box o (foxo4) acetylation mediates apoptosis of podocytes in diabetes mellitus. *PLoS ONE*.

[3] Kvansakul M., Hinds M. G. (2015). The Bcl-2 family: structures, interactions and targets for drug discovery. *Apoptosis*.

